# Interobserver and Intraobserver Variability of Four Suprascapular Notch Classification Systems

**DOI:** 10.7759/cureus.54389

**Published:** 2024-02-18

**Authors:** Christos Yiannakopoulos, Elina Gianzina, Spilios Dellis, Georgios Kalinterakis, Iakovos Vlastos, Konstantinos Mastrantonakis

**Affiliations:** 1 Orthopaedics, IASO Hospital, Athens, GRC; 2 School of Physical Education & Sport Science, National and Kapodistrian University of Athens, Athens, GRC; 3 Orthopaedics and Traumatology, Rethymno General Hospital, Rethymno, GRC

**Keywords:** interobserver variability, intraobserver variability, suprascapular notch, suprascapular neuropathy, morphology, computed tomography, classification systems, anatomy

## Abstract

Introduction: Knowledge of the morphology of the suprascapular notch is clinically beneficial in patients with suspected suprascapular nerve compression or palsy. Several classification systems have been proposed for the morphological classification of the suprascapular notch and its several anatomical variations. The purpose of this study was to evaluate the inter- and intraobserver reliability of four different classification systems for suprascapular notch typing analysing shoulder computed tomography (CT) scans.

Methods: Shoulder CT scans from 109 subjects (71.5% males) were examined by three raters of various experience levels, one senior, one experienced, and one junior orthopaedic surgeon. The CT scans were evaluated quantitatively and qualitatively and the suprascapular notch was classified according to four classification systems at two separate timepoints, four weeks apart. To determine consistency among the same or different raters, the Kappa statistic was performed and intrarater reliability for each rater between the first and the second evaluation was assessed using Cohen’s kappa. Reliability across all raters at each timepoint was assessed using the Fleiss kappa.

Results: Agreement was almost perfect for all the classification systems and amongst all raters, regardless of their experience level. There were no significant differences between the raters on any of the evaluations. The overall interobserver agreement for all classifications was almost perfect.

Conclusion: The four suprascapular notch classification systems are reliable, and the rater's experience level has no impact on the evaluation.

## Introduction

The suprascapular notch (SSN) is a bony depression located at the superior border of the scapula, at the base of the coracoid process [1]. The SSN is converted to a foramen or a canal by the superior transverse scapular ligament (STSL) which passes over the notch and attaches to the base of the coracoid process [1]. The suprascapular nerve (SSn) arises from the upper trunk of the brachial plexus and passes through the notch, entering the supraspinatus fossa [1,2]. It then gives off motor branches to the supraspinatus and infraspinatus muscles and sensory fibres to the glenohumeral joint and the skin of the posterior surface of the shoulder [2]. The suprascapular artery passes typically over the STSL and the suprascapular vein through the notch with SSn [1].

The bony morphology of the SSN is subject to significant variability [3] and sexual dimorphism [4]. Reduction of the space available for the suprascapular nerve has been implicated in the pathogenesis of SSn neuropathy, a type of peripheral nerve entrapment neuropathy [5,6]. In patients with a V-shaped notch or an ossified STSL entrapment of the SSn is more probable [6]. Knowledge of the SSN morphology is thus significant in the evaluation of patients with possible SSn neuropathy. Identifying the anatomical variations of the SSN is important for the management of associated clinical disorders such as shoulder pain that can result from entrapment of the SSn within a narrow suprascapular foramen or below the SSTL in the absence of a foramen [7]. The most efficient modality for imaging of cortical bone and the osseous morphology in vivo is computed tomography (CT) with three-dimensional (3D) reconstruction [8]. CT is fast, readily available, low-cost, and offers high resolution, although exposure to ionizing remains a significant concern. Additionally, three-dimensional (3D) reconstruction allows multiplanar and volumetric reconstructions [9,10].

Various classification systems attempted to describe the variation in SSN morphology with visual inspection of dry human scapulae [11-13]. Rengachary et al. [11] modified the classification of Hrdicka [12] and described six types of SSN, while Natsis et al. [13] described five types of SSN. Polgul et al. [14], using measurements of the SSN depth and width, modified the Natsis et al. [13] classification and described five types of SSN. Ticker et al. [15] classified the suprascapular notch as either “U”- or “V”-shaped, evaluating separately the degree of ossification of the TSL. Iqbal et al. [16] reported three types of SSN, namely U-shaped (13.2%), V-shaped (20%), and J-shaped (22%), and Zhang et al. [17] described seven SSN types in the Chinese population.

Dunkelgrun et al. [18] reported the inter- and intraobserver reliability of the Rengachary et al. [11] and the Ticker et al. [15] SSM classification. They used a sample of 623 scapulae from 322 individuals and showed only moderate agreement for both classifications and substantial only for the superior border of the notch. To the best of our knowledge, there are no reports on the inter- and intraobserver reliability of SSN classifications using 3D CT scans or visual examination of dry scapulae.

This study aimed to evaluate the interobserver and intraobserver reliability of the Rengachary et al. [11], Natsis et al. [13], Polgul et al. [14], and Zhang et al. [17] classification systems for suprascapular notch typing analysing CT scans of the shoulder.

## Materials and methods

We collected 109 shoulder CT scans obtained due to shoulder trauma prior to the operation. Patients with previous shoulder trauma, rotator cuff tear, shoulder osteoarthritis and/or operations, or neurological symptoms related to the brachial plexus or the suprascapular nerve more specifically were excluded. This was a retrospective study and only the sex and age were revealed, and thus informed consent was not required. The study was approved by the Institutional Scientific Committee IASO Hospital (approval number 2023-01499). The mean age of the study sample was 59.6±12.8 years, 78 (71.5%) were males, and the right side was involved in 61 cases (56%). Three raters of various experience levels, one senior, one experienced, and one junior orthopaedic surgeon evaluated the CT scans at two separate timepoints, four weeks apart, and classified the SSN according to the four classification systems. Before the assessment, the raters received a copy of all original publications along with written instructions for performing the evaluation. The raters were asked to evaluate the shape of the SSN and performed measurements on 3D CT scans to complete the evaluation. All Digital Imaging and Communications in Medicine (DICOM) files were processed using Picture Archiving and Communication Systems (PACS) with a dedicated 3D analysis software (syngo.via RT Image Suite; Siemens Healthineers, Erlangen, Germany).

Suprascapular notch classification systems

**Figure 1:**
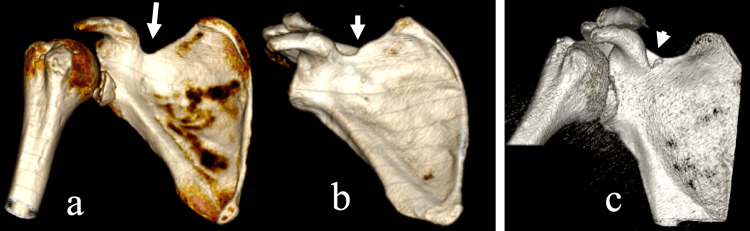
Common morphological types of the suprascapular notch as seen on 3D-CT models. Wide depression of the superior border forms of the (a) scapula (long arrow), (b) U-type (short arrow), and (c) V-type (arrowhead).

**Figure 2:**
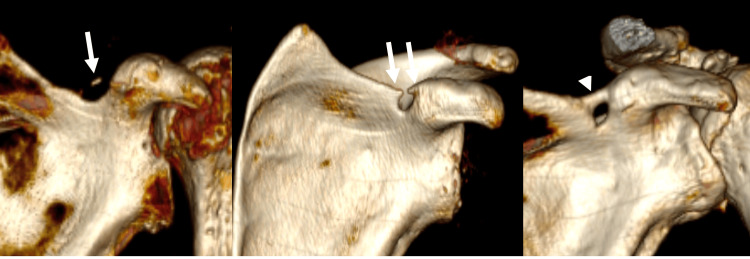
3D-CT models show variants of superior transverse scapular ligament ossification: (a) Mid-ligament ossification (long arrow), (b) medial and lateral ossification (double arrows), and (c) complete ossification (arrowhead)

The suprascapular notch has been classified into various types based on anatomical observations and measurements on dry cadaveric scapulae [11-14] and CT scans [6]. Common morphological types of the suprascapular notch are shown in Figures 1, 2.

In the current study, we evaluated four classifications of the SSN, i.e. the Rengachary et al. [11], Natsis et al. [13], Polgul et al. [14] and Zhang et al. [17] classifications. The details of the classifications are presented in Table 1. 

**Table 1:** The four suprascapular notch classification systems evaluated in this study

Classification System	Type I	Type II	Type III	Type IV	Type V	Type VI	Type VII
Rengachary et al. [11]	Absent suprascapular notch. The superior border of the suprascapular notch forms a wide depression from the medial angle of the scapula to the coracoid process, 8%	V-shaped suprascapular notch, located in the middle third of the superior border of the scapula, 31%	U-shaped suprascapular notch with nearly parallel margins, 48%	Similar to type II, but very small; the suprascapular nerve often passes through a groove next to the suprascapular notch, 3%	Similar to type III, but very small, with a partially ossified Transverse Scapular Ligament, 6%	The transverse scapular ligament, is completely ossified and the suprascapular notch is converted to a foramen, 4%	-
Natsis et al. [13]	No discrete suprascapular notch, 8.3%	The suprascapular notch is longest in its transverse diameter, 41.85%	The suprascapular notch is longest in its vertical diameter, 41.85%	Bony foramen, 7.3%	Presence of a suprascapular notch and a bony foramen, 0.7%	-	-
Polgul et al. [14]	The suprascapular notch has longer maximal depth than superior transverse diameter, 26%; Subtype IA: middle transverse diameter>superior transverse diameter; Subtype IB: middle transverse diameter < superior transverse diameter	The suprascapular notch has equal maximal depth, superior transverse diameter and middle transverse diameter, 3%	The superior transverse diameter is longer than the maximal depth, 57.6%;Subtype IIIA: middle transverse diameter>superior transverse diameter; Subtype IIIB: middle transverse diameter < superior transverse diameter	The suprascapular notch is a bony foramen, 7.4%	No discrete notch, 6%	-	-
Zhang et al. [17]	Square root type, √,44.8%	U type, 41.9%	V type, 6.2%	O type, 2.9%	Reverse omega or horseshoe type, Ʊ, 1.9%	W type, 1.6%	Double O type, 0.6%

Statistical analysis

To determine consistency among the same or different raters the Kappa statistic was performed. To assess the reliability of the classification systems, power analysis was performed a priori to calculate the minimum sample size of the number of shoulder CT scans that needed to be reviewed by the raters. To test a kappa of 0.5 (moderate agreement) for an outcome with five categories, with an alpha value of 0.05, a beta value of o0.20, and three raters, a minimal sample size of 71 was necessary. Intrarater reliability for each rater between the first and the second evaluation was assessed using Cohen’s kappa. Reliability across all raters at each timepoint was assessed using the Fleiss kappa. The 95% confidence interval (CI) was calculated for significance. The strength of agreement was categorized according to Landis and Koch [19]. A κ value <0.00 was rated poor; 0.00-0.20, slight; 0.21-0.40, fair; 0.41-0.60, moderate;0.61- 0.80, substantial; and 0.81-1.00, almost perfect. Statistical analysis was performed using IBM SPSS Statistics for Macintosh, Version 28.0 (Released 2021; IBM Corp., Armonk, New York, United States).

## Results

Intraobserver agreement between the first and the second evaluation is presented in Table 2. Agreement was almost perfect for classifications and all raters, regardless of their experience level. There were no significant differences between the raters on any of the evaluations.

**Table 2:** Report of the intraobserver agreement. Intraobserver agreement between the first and the second evaluation of each rater. Kappa values±Standard Error are presented.

	Rengachary et al. [11]	Natsis et al. [13]	Polguj et al. [14]	Zhang et al. [17]	p-value
Rater 1	0.897±0.037	0.895±0.038	0.829±0.048	0.798±0.046	< 0.001
Rater 2	0.899±0.037	0.941±0.029	0.796±0.054	0.838±0.043	< 0.001
Rater 3	0.874±0.040	0.871±0.041	0.802±0.052	0.859±0.040	< 0.001

Interobserver agreement as evaluated by the Fleiss Multirater Kappa for the Rengachary et al. [11] classification was 0.887±0.035 (95%CI 0.819-0.956), for the Natsis et al. [13] classification, it was 0.887±0.040 (95%CI 0.808-0.966), for the Polguj et al. [14] classification, it was 0.831±0.043 (95%CI 0.746-0.917), and for the Zhang et al. [17] classification, it was 0.838±0.032 (95%CI 0.774-0.902). The overall interobserver agreement for all classifications was almost perfect.

## Discussion

This study evaluated the inter- and intraobserver agreement of four SSN classification systems between raters of varying experience level. Overall, there was excellent inter- and intraobserver agreement between the raters regardless of their experience level. Although the type and area of the suprascapular notch are considered predisposing factors for suprascapular nerve neuropathy, it is associated in 42% of the cases with solitary paralabral cysts, which are related with the presence of shoulder superior labrum superior labral anterior to posterior (SLAP) lesions [20]. Honoki et al. studied the 3D CT images in 53 patients with SSn palsy and compared the morphology of the SSN with the morphology in a series of 1010 patients without SSn palsy [21]. They did not find an association between narrow SSN or ossification of the STSL with SSn palsy [21].

Specific abnormalities of the SSN are thought to be among the major causes of suprascapular neuropathy, and in particular the presence of an ossified or bifid STSL and variations such as the double suprascapular foramen, and a narrowed "V" shape configuration [4,11,22]. Knowledge of the SSN morphology in patients with posterior shoulder pain and suspected SSn peripheral neuropathy is very important in the diagnosis and treatment of the problem [5,6,11,20,21].

The five-type classification of Natsis et al. [13] was based on vertical and transverse diameter SSN measurements, while Polguj et al. [14] used three similar measurements, maximal depth, superior and middle transverse diameter, and added one more type of SSN in the Natsis et al. [13] classification. In the Polgul et al. classification, the addition of subtypes in types I and III, as well as the addition of type II with equal depth on both transverse diameters may lead to observer disagreement, especially when the differences between the measurements are modest [14]. There were no cases with type VI (W type) and VII (double 0) SSN according to the Zhang et al. [17] classification and no cases with Natsis et al. [13] type V SSN (notch and bony foramen) in our study population.

There are several potential sources of error in the SSN classifications. In the Rengachary et al. classification, a type IV notch, which is characterized by a small V shape, can easily be confused with type III [11]. For example, in the paper by Inoue et al., there is a rather minor morphological difference between types III and IV [3]. Additionally, there is no clear definition to discriminate a type V SSN with a partially ossified suprascapular ligament. It is not uncommon to see traction osteophytes at the superior opening of the SSN at the attachment sites of the STSL. Ossification of the STSL seems to be an age-related process [3,21,23]. The quantitative classification by Polguj et al. depends on the accurate measurement of the vertical and horizontal dimensions of the SSN and is prone to measurement bias, especially in Type II, where all three measurements must be equal [14]. In the Zhang et al. classification, there is occasionally difficulty in the discrimination between types II and II [17].

Dunkelgrun et al. [18] examined the inter- and intraobserver reliability of the Rengachary et al. [11] and the Ticker et al. [15] classifications. The mean kappa value for the Rengachary classification was 0.468 and for the Ticker classification was 0.531 for the inferior border of the notch and 0.736 for the superior border of the notch [18]. The Ticker classification accepts only two SSN types, a U-type, and a V-type, leaving many SSN types unclassified [15]. Additionally, Dunkelgrun et al. used digital images and analysis software to perform measurements and to classify the SSN without reporting agreement or variation between the measurements [18]. In the current study, the raters were experienced in performing measurements on 3D CT scans and in the classification of the SSN and additionally, there was no time limit in the evaluation of the notch types.

The present study has several limitations. The classification of the SSN was performed on CT scans although the original classifications were described on dry scapulae. The CT scans were obtained from patients with shoulder trauma primarily but without symptoms of SSn neuropathy. The morphology of the STSL was not possible to be taken into consideration using 3D CT scans, although the ligament occupies a significant part of the SSN. Additionally, rare subtypes of the suprascapular notch were not evaluated due to the limited number of our cases.

Future studies using high-resolution MRI may be able to evaluate the dimensions of the STSL and the thickness of the suprascapular nerve. Additionally, MRI performed in various shoulder positions such as the Abduction and External Rotation (ABER) position may be helpful in the evaluation of the scapula kinematics and the mobility of the suprascapular nerve.

## Conclusions

The current study showed that the morphology of the suprascapular notch can readily be evaluated in vivo using 3D CT scanning. Additionally, and for the first time in the literature, four suprascapular notch evaluation systems have been evaluated and proved to be reliable, while the experience level of the various raters had no impact on the evaluation. The possible compression effect of the various suprascapular notch types on the suprascapular nerve remains to be further evaluated. A comparison of the occurrence of the various suprascapular notch types in patients with proven suprascapular nerve entrapment would be clinically very useful.
